# A step beyond the hygiene hypothesis—immune-mediated classes determined in a population-based study

**DOI:** 10.1186/s12916-019-1311-z

**Published:** 2019-04-09

**Authors:** Vladeta Ajdacic-Gross, Margot Mutsch, Stephanie Rodgers, Anja Tesic, Mario Müller, Erich Seifritz, En-Young N. Wagner, Roland von Känel, Markus A. Landolt, Nina Steinemann, Viktor von Wyl, Enrique Castelao, Marie-Pierre F. Strippoli, Jennifer Glaus, Caroline Vandeleur, Pedro M. Marques-Vidal, Peter Vollenweider, Martin Preisig

**Affiliations:** 1 Department of Psychiatry, Psychotherapy and Psychosomatics, Psychiatric Hospital, University of Zurich, PO Box 2019, CH-8021 Zurich, Switzerland; 2 Epidemiology, Biostatistics and Prevention Institute, University of Zurich, Zurich, Switzerland; 3 Department of Consultation-Liaison Psychiatry and Psychosomatic Medicine, University Hospital, Zurich, Switzerland; 4 University Children’s Hospital Zurich and Children’s Research Center, Zurich, Switzerland; 5 Division of Child and Adolescent Health Psychology, Department of Psychology, University of Zurich, Zurich, Switzerland; 6 Department of Psychiatry, Center for Research in Psychiatric Epidemiology and Psychopathology, Lausanne University Hospital, Prilly, Switzerland; 7 Genetic Epidemiology Research Branch, Intramural Research Program, National Institute of Mental Health, Bethesda, MD USA; 8 CHUV Lausanne, University of Lausanne, Lausanne, Switzerland

**Keywords:** Immune system, Latent class analysis, Hygiene hypothesis, Chronic diseases, Mental disorders, Biomarkers

## Abstract

**Background:**

Comorbidity patterns of childhood infections, atopic diseases, and adverse childhood experiences (ACE) are related to immune system programming conditions. The aim of this study was to make a step beyond the hygiene hypothesis and to comprehensively classify these patterns with latent class analysis (LCA). A second aim was to characterize the classes by associations with immunological, clinical, and sociodemographic variables.

**Methods:**

LCA was applied to data from the CoLaus|PsyCoLaus study (*N* = 4874, age range 35–82 years) separately for men and women. It was based on survey information on chickenpox, measles, mumps, rubella, herpes simplex, pertussis, scarlet fever, hay fever, asthma, eczema, urticaria, drug allergy, interparental violence, parental maltreatment, and trauma in early childhood. Subsequently, we examined how immune-mediated classes were reflected in leukocyte counts, inflammatory markers (IL-1β, IL-6, TNF-α, hsCRP), chronic inflammatory diseases, and mental disorders, and how they differed across social classes and birth cohorts.

**Results:**

LCA results with five classes were selected for further analysis. Latent classes were similar in both sexes and were labeled according to their associations as neutral, resilient, atopic, mixed (comprising infectious and atopic diseases), and ACE class. They came across with specific differences in biomarker levels. Mental disorders typically displayed increased lifetime prevalence rates in the atopic, the mixed, and the ACE classes, and decreased rates in the resilient class. The same patterns were apparent in chronic inflammatory diseases, except that the ACE class was relevant specifically in women but not in men.

**Conclusions:**

This is the first study to systematically determine immune-mediated classes that evolve early in life. They display characteristic associations with biomarker levels and somatic and psychiatric diseases occurring later in life. Moreover, they show different distributions across social classes and allow to better understand the mechanisms beyond the changes in the prevalence of chronic somatic and psychiatric diseases.

## Introduction

Dense, complex comorbidity networks—diseasomes [1]—exist both within and across chronic inflammatory diseases, neurodevelopmental/mental disorders, and, as well, between them and infections and atopic diseases. A systemic understanding of comorbidity networks will open new avenues to approach the underlying pathological mechanisms. This study focused on comorbidity patterns of infectious and atopic diseases usually emerging early in life. Concepts related to the immune system programming [2] or to the neonatal window of opportunity [3] suggest that these comorbidity patterns reflect characteristic, lasting, and far-reaching imbalances in the immune system.

Epidemiological research has already addressed immune system programming from different perspectives. The hygiene [4] or, rather, the “Old Friends” hypothesis [5] has postulated that continuing or frequent low-level exposure to a variety of microbial and helminthic pathogens (the “Old Friends”) in infancy and early childhood boosts the immune system while at the same time improves its capacity for inflammation control. Modern improvements in hygiene levels are believed to have changed children’s immune system programming patterns and to have contributed to the increasing rates of atopic diseases. In the same vein, the early development of a healthy microbiota has been shown to be protective against atopies [6–8] and to be associated with mental health across the life course [9].

In addition, adverse childhood experiences (ACE) have been consistently shown to increase the risk for neurodevelopmental/mental disorders [10–13] and for somatic diseases [14] across the life course. The proposed mechanisms include neuroendocrine [15] as well as immune processes [16], both with the potential to modify immune system programming in childhood [17–19] and to seriously impact somatic and mental health [20, 21].

The first aim of this study was to develop an integrated approach to immune system programming by classifying comorbidity patterns of childhood infections and atopic diseases together with ACE. The statistical model was latent class analysis (LCA); the latent classes will also be referred to as immune-mediated classes. The second aim was to further characterize these immune-mediated classes by white blood cell (WBC) counts and inflammatory markers as well as their association patterns with other groups of diseases and sociodemographic variables. The analyses were stratified by sex. The database came from CoLaus|PsyCoLaus, a large epidemiological study in Switzerland.

## Methods

### The CoLaus|PsyCoLaus cohort

CoLaus|PsyCoLaus [22, 23] is a prospective cohort study designed to study cardiovascular risk factors (CoLaus) and mental disorders (PsyCoLaus) in the community and to determine their associations. The baseline investigation was carried out between 2003 and 2006. For the allocation of the initial CoLaus sample, the subjects were randomly selected among the 35- to 75-year-old residents of the city of Lausanne (Switzerland) according to the civil register (*n* = 6734). The survey was conducted in the French language. Participation in the psychiatric evaluation was first confined to 35- to 67-year-olds of the CoLaus sample; 67% of subjects within this age range (*n* = 3720) agreed to participate in the PsyCoLaus part.

**Fig. 1:**
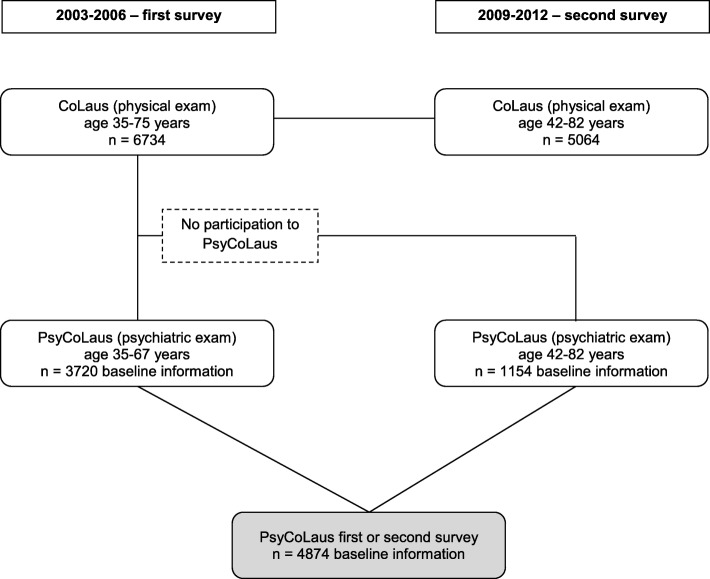
CoLaus|PsyCoLaus sample design

The first follow-up of CoLaus|PsyCoLaus was conducted between 2009 and 2012. Information on physical health was obtained from 5064 (75%) of the *n* = 6734 participants. In the PsyCoLaus subsample, 87% remained in the study, i.e., 3188 out of the 3673 participants. Participants who had missed the psychiatric part of the baseline examination (including those initially aged 67–75 years) were given the opportunity to complete it at follow-up. Therefore, the sample comprising baseline data from both interviews increased by *n* = 1154 and summed to *n* = 4874 (see overview in Fig. 1).

### The Diagnostic Interview for Genetic Studies

The main survey instrument of PsyCoLaus was the French version of the semi-structured Diagnostic Interview for Genetic Studies (DIGS) [24, 25]. The DIGS collects information related to common mental disorders and returns lifetime prevalence information. It includes also data related to the course and chronology of comorbid features, somatic conditions, and a range of developmental and psychosocial risk factors [22]. A French version [24] of the DIGS [25] was used to assess diagnostic information on mental disorders (see below).

The DIGS collects information on a broad spectrum of the Diagnostic and Statistical Manual of Mental Disorders IV (DSM-IV) Axis I criteria related to mental disorders and, moreover, on the course and chronology of comorbid features [25]. The brief phobia section of the DIGS was replaced by the corresponding, more extensive, sections of the Schedule for Affective Disorders and Schizophrenia—Lifetime and Anxiety Disorder Version (SADS-LA) [26]. This elicited detailed information related to the DSM-IV criteria for agoraphobia with or without panic attacks, social phobia, and specific phobias. The DIGS and the SADS-LA elicit lifetime diagnoses of common mental disorders. Successful inter-rater and test-retest reliability of the French version of the DIGS have been established for major mood and psychotic disorders [24] as well as for substance use and antisocial personality disorders [27]. Similarly, for the anxiety sections of the French version of the SADS-LA, inter-rater and test-retest reliability are good [28]. In this analysis, we grouped mental disorders into five groups: neurodevelopmental, early-onset anxiety (average age of onset up to 12), late-onset anxiety (average age of onset above 12), mood, and substance use disorders (see notes to Table 3).

The information on lifetime diagnosis of infections and somatic conditions was derived using an extended version of the medical history parts of the DIGS and an early version of the Schedule for Affective Disorders and Schizophrenia—Lifetime and Anxiety Disorder Version (SADS-LA) [26] and was based on reporting of the participants. The questions were related to ever having been diagnosed with various infections, diseases of the nervous system, and cardiovascular, respiratory, gastrointestinal, metabolic, and dermatological conditions as well as allergies and hormonal problems. The lifetime prevalence of migraine was assessed according to the criteria of the International Classification of Headache Disorders (ICHD-II) [29], using the validated French version of the Diagnostic Interview for Headache Syndromes (DIHS). For the present analysis, infections (chickenpox, measles, mumps, rubella, scarlet fever, pertussis, herpes labialis), atopic diseases (allergic asthma, other asthma, allergic eczema, other eczema, hay fever, urticaria, drug allergy), chronic inflammatory diseases (ulcer, irritable bowel syndrome, cystitis, acne, psoriasis, plus migraine), and mental disorders were selected. The age range of the sample implies that very few participants had received any measles-mumps-rubella vaccine in childhood, as measles and other vaccination schedules were only introduced by Swiss health authorities in the 1960s and 1970s and were used by a minority of children at the beginnings.

Family-related ACE were represented by the following questions (variables):

- Did your parents fight with each other frequently? (interparental violence)
- Did your parents ever do anything that frightened you (like lock you in a closet)? (fear of maltreatment)

In addition, the information about traumatic experiences in childhood was extracted from the DIGS section on posttraumatic stress disorder. Potentially traumatizing events comprised accidents, physical assaults, witnessing murder, violence or death by accident, sexual abuse, and, without relevance for the present study, combat and/or war. The age before which the event must have occurred was 10 years. Socioeconomic status was assessed using the Hollingshead index [30].

### Inflammatory markers and WBC counts

The biomarkers analyzed in this study comprised white blood cells (WBC) and inflammatory markers (interleukin-1β (IL-1β), interleukin-6 (IL-6), tumor necrosis factor-α (TNF-α), and high-sensitivity C-reactive protein (hsCRP)). WBC and inflammatory markers were assessed in the baseline examination (PsyCoLaus subsample *n* = 4671) and the inflammatory markers also in the first follow-up (*n* = 4057).

Morning venous blood samples were taken from participants without current infection and allowed to clot. For the cytokine measurements, serum was preferred to plasma, as it has been shown that different anticoagulants may differentially affect absolute cytokine levels [31]. Serum samples were stored at − 80 °C before assessment and sent on dry ice to the laboratory. Cytokine levels were measured using a multiplexed particle-based flow cytometric cytokine assay [32]. Good agreement between signal and cytokine was found within the assay range (*R*^2^ ≥ 0.99). Lower detection limits (LOD) were 0.2 pg/ml. For concentrations below the LOD, a value of 0.1 pg/ml was assigned.

hsCRP was assessed during baseline and follow-up physical evaluations using immunoassay and latex HS (IMMULITE 1000-High, Diagnostic Products Corporation, LA, CA, USA), with maximum intra- and interbatch coefficients of variation of 1.3% and 4.6%, respectively [23]. Subjects with a hsCRP level higher than 10 mg/l were excluded as such an elevation is likely to be attributable to acute infection.

WBC counts were performed on an XN-2100 apparatus (Sysmex, Horgen, Switzerland) during the first follow-up of CoLaus. Data was available for 2963 participants. Participants with/without WBC counts did not differ as to sex and age; however, the latter had a slightly lower SES score (3.29 vs. 3.41, *p* = 0.002). Neutrophils, lymphocytes, monocytes, eosinophils, and basophils were represented as proportions of total leukocytes. The WBC variables were smoothed (square root, log)—if appropriate—in order to improve the statistical parameters of the distributions.

In order to combine values from the baseline and the follow-up measurement, the inflammatory marker values were transformed to ranks. In a preliminary step, the ranks were calculated for the participants with both measurements (by sex and separately for each measurement). In the next step, participants with only one measurement were given the ranks of their neighbors. If ranks of both measurements were available, the mean rank was used, otherwise the rank of the available measurement.

### Statistical analysis

LCA has been used in comorbidity analyses both in somatic medicine [33–37] and psychiatric research [38–40]. LCA is a person-centered approach to classification, i.e., it aims to group individuals into homogeneous classes [41, 42]. The classes represent subgroups of individuals based on similar responses and characteristics.

The LCA in this study was based on variables representing infectious childhood diseases (chickenpox, measles, mumps, rubella, herpes simplex, pertussis, scarlet fever), atopic diseases (hay fever, asthma, eczema, urticaria, drug allergy), and childhood adversities (interparental violence, parental maltreatment, trauma in early childhood). LCA was conducted using Mplus version 7 [43]. To avoid problems with local maxima, the number of random starts was set to 2500 for the first step, using the 250 best solutions in the final calculation. One to six latent class models were routinely fitted to the data in order to determine the optimal number of latent classes in the final model. We considered several fit indices: the Akaike information criterion (AIC), the Bayesian information criterion (BIC), and the sample size-adjusted BIC (ABIC) and in addition the Lo-Mendell-Rubin likelihood ratio test (LMR-LRT) [44]. Typically, we prefer models with a number of classes between the number suggested by the BIC and the number suggested by the AIC. The model selection is furthermore determined by the distinction between the classes, their size, and their theoretical adequacy.

In further analyses, we applied cross-tabulations, analysis of variance (ANOVA), general linear models, Kruskal-Wallis test, and logistic regression models. Programming and this part of the analyses were carried out with SPSS Statistics (version 23). We explicitly did not perform adjustment for multiple testing for several reasons. First of all, there are two technical reasons:

- Adjustment for multiple testing is appropriate under specific conditions including homogeneous items (as well as large *N*s, precise measurements, homogeneous conditions, etc.); in contrast, the diseases/disorders we are investigating subsume heterogeneous conditions and comprise different subtypes; this applies not only to grouped disorders but similarly also to most of the specific disorders or diseases comprised in this study; one part of the analysis strategy is to prioritize the *β*-error over the *α*-error, that is, to increase the probability to account for or to detect minor subtypes
- Another part of the analysis strategy is to focus on pattern recognition, explicitly through the LCA in this study, but also implicitly in analyses of groups of markers and disorders; pattern recognition is prioritized over analysis of single conditions; adjustment for multiple testing is uncommon in any pattern recognition analysis such as involving LCA, factor analysis, and correspondence analysis (see also [45])

Beyond these reasons, there are methodological pros and cons whether statistical precision could be helpful in crunching complex issues. We assume that complexity adapted modeling strategies are currently more urgently needed.

## Results

Table 1 shows the sociodemographic and socioeconomic characteristics of the sample. The variables used in conducting the LCAs (infections, atopies, ACE) are displayed in Table 1 with frequencies and univariate distributions. Similarly, outcome variables (chronic inflammatory diseases, neurodevelopmental/mental disorders) are displayed in Table 2, whereas the WBC counts and the inflammatory markers are summarized in Table 3.

**Table 1:** Infections, atopies, and adverse childhood experiences in CoLaus|PsyCoLaus: frequencies and proportions

	All	Men	Women
Infections			
Chickenpox	3733 (83.3%)	1657 (80.8%)	2076 (85.5%)
Measles	3323 (75.7%)	1442 (72.0%)	1881 (78.8%)
Mumps	2720 (60.8%)	1291 (62.9%)	1429 (59.1%)
Rubella	283 (5.3%)	62 (2.7%)	221 (8.5%)
Scarlet fever	216 (4.4%)	80 (3.5%)	136 (5.2%)
Pertussis	377 (7.7%)	113 (5.0%)	264 (10.1%)
Herpes labialis	767 (15.7%)	260 (11.5%)	507 (19.4%)
Atopies			
Atopic asthma	291 (6.0%)	107 (4.7%)	184 (7.0%)
Other asthma	373 (7.7%)	141 (6.2%)	232 (8.9%)
Atopic eczema	230 (4.7%)	72 (3.2%)	158 (6.1%)
Other eczema	314 (6.4%)	125 (5.5%)	189 (7.2%)
Hay fever	890 (18.3%)	420 (18.6%)	470 (18.0%)
Urticaria	179 (3.7%)	59 (2.6%)	120 (4.6%)
Drug allergy	493 (10.1%)	164 (7.2%)	329 (12.6)
Adverse childhood experiences			
Fights among parents	609 (12.6%)	264 (11.8%)	345 (13.4%)
Fear of parental maltreatment	464 (9.6%)	191 (8.5%)	273 (10.5%)
Trauma below age of 10	189 (3.9%)	56 (2.5%)	133 (5.1%)

**Table 2:** Chronic inflammatory diseases and main groups of neurodevelopmental/mental disorders in CoLaus|PsyCoLaus: frequencies and proportions

	All	Men	Women
Chronic inflammatory diseases			
Ulcer	265 (5.4%)	136 (6.0%)	129 (4.9%)
Irritable bowel syndrome	188 (3.9%)	52 (2.3%)	136 (5.2%)
Cystitis	1069 (21.9%)	116 (5.1%)	953 (36.5%)
Acne	475 (9.7%)	191 (8.4%)	284 (10.9%)
Psoriasis	220 (4.5%)	108 (4.8%)	112 (4.3%)
Migraine	664 (13.6%)	196 (8.7%)	468 (17.9%)
Neurodevelopmental/mental disorders^1^			
Neurodevelopmental disorders	353 (7.2%)	214 (9.5%)	139 (5.3%)
Early anxiety disorders	1104 (22.7%)	377 (16.7%)	727 (27.9%)
Late anxiety disorders	655 (16.2%)	233 (10.3%)	422 (16.2%)
Mood disorders	2161 (44.3%)	770 (34.0%)	1391 (53.3%)
Substance disorders	645 (13.2%)	488 (21.6%)	157 (6.0%)

^1^Neurodevelopmental disorders: tics, attention-deficit hyperactivity disorder (ADHD), conduct disorder (CD), oppositional defiant disorder (ODD); early anxiety disorders: separation anxiety disorder, overanxious disorder, specific phobias (animals), social phobia; late anxiety disorders: generalized anxiety disorder (GAD), panic, agoraphobia, specific phobias (excl. animals); mood disorders: major depression disorder, dysthymia, bipolar disorders; substance disorders: alcohol, cannabis, other illicit drug abuse/dependence

**Table 3:** WBC counts and inflammatory markers in CoLaus|PsyCoLaus (mean and 95% confidence interval)

	All	Men	Women
White blood cell counts^1^			
Total leukocytes (in 1000/mm^3^)	6.25 (6.19–6.32)	6.40 (6.30–6.49)	6.13 (6.05–6.21)
Neutrophils %	0.548 (0.545–0.551)	0.545 (0.540–0.550)	0.550 (0.546–0.555)
Basophils %	0.0058 (0.0057–0.0059)	0.0056 (0.0054–0.0057)	0.0060 (0.0059–0.0062)
Eosinophils %	0.0294 (0.0288–0.0301)	0.0316 (0.0307–0.0326)	0.0276 (0.0268–0.0284)
Lymphocytes %	0.326 (0.323–0.329)	0.321 (0.317–0.326)	0.330 (0.326–0.334)
Monocytes %	0.0868 (0.0860–0.0876)	0.0924 (0.0912–0.0936)	0.0821 (0.0812–0.0831)
Inflammatory markers at baseline			
IL-6 (pg/ml)	8.15 (6.46–9.84)	8.11 (5.55–10.67)	8.19 (5.93–10.44)
IL-1β (pg/ml)	4.25 (3.49–5.02)	4.31 (3.38–5.24)	4.20 (3.03–5.38)
TNF-α (pg/ml)	6.62 (4.95–8.29)	5.52 (4.37–6.66)	7.57 (4.62–10.53)
hsCRP (mg/l)	1.81 (1.76–1.87)	1.73 (1.65–1.80)	1.89 (1.81–1.97)
Inflammatory markers at follow-up 1			
IL-6 (pg/ml)	17.54 (15.39–19.69)	17.53 (14.45–20.60)	17.56 (14.56–20.55)
IL-1β (pg/ml)	4.45 (3.84–5.06)	4.53 (3.81–5.24)	4.39 (3.45–5.34)
TNF-α (pg/ml)	10.46 (8.44–12.47)	10.08 (8.57–11.59)	10.78 (7.27–14.29)
hsCRP (mg/l)	1.89 (1.83–1.95)	1.77 (1.69–1.85)	1.98 (1.90–2.06)

^1^WBC counts were assessed only at follow-up 1

### Immune-mediated classes derived from LCA

In LCA of infections, atopic diseases, and ACE, the five-class solution emerged for both sexes as a reasonable choice balanced in respect of the fit indices (see Table 4), with a clear substantial differentiation between the classes and sufficient class sizes, and corroborated by differentiated association patterns (see below). Compared to the four-class solution, the five-class solution added the mixed class. The six-class solution was burdened by classes with very small sizes.

**Table 4:** Model fit indices for latent class analyses with one through six classes, men and women

Fit statistics	1-class model	2-class model	3-class model	4-class model	5-class model	6-class model
Men						
AIC	21,775.3	21,055.2	20,801.7	20,639.2	20,604.3	20,599.4
BIC	21,872.7	21,255.5	21,105.1	21,045.7	21,113.8	21,211.9
ABIC	21,818.6	21,144.3	20,936.7	20,820.1	20,831.1	20,872.0
LMR-LRT. adj.		750.8	287.4	197.0	70.4	40.6
*p*-value		< 0.001	< 0.001	0.052	0.648	0.016
Women						
AIC	30,328.7	29,729.7	29,466.4	29,262.3	29,186.2	29,148.4
BIC	30,428.4	29,935.1	29,777.4	29,678.9	29,708.3	29,776.2
ABIC	30,374.4	29,823.8	29,609.0	29,453.3	29,425.6	29,436.2
LMR-LRT. adj.		630.5	297.2	238.4	111.4	73.2
*p*-value		< 0.001	< 0.001	0.003	0.002	0.106

*AIC* Akaike information criterion, *BIC* Bayesian information criterion, *ABIC* sample size-adjusted Bayesian information criterion, *LMR-LRT. adj.* Lo-Mendell-Rubin likelihood ratio test—adjusted

**Fig. 2:**
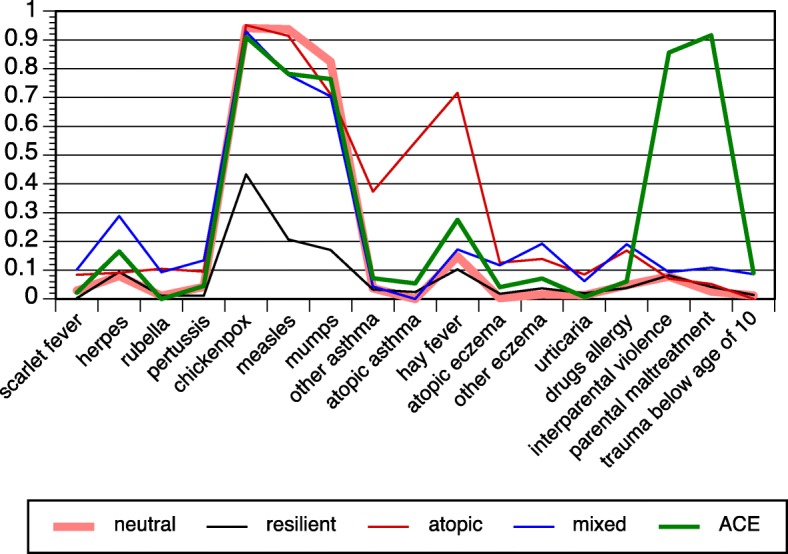
Latent class analysis with infectious childhood diseases, atopic diseases, and childhood adversities in the PsyCoLaus study; probabilities of the five-class model, men

**Fig. 3:**
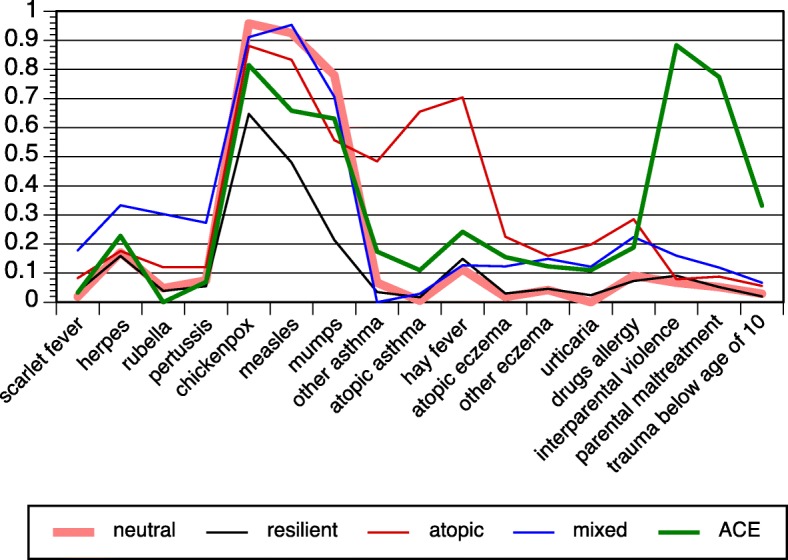
Latent class analysis with infectious childhood diseases, atopic diseases, and childhood adversities in the PsyCoLaus study; probabilities of the five-class model, women

**Fig. 4:**
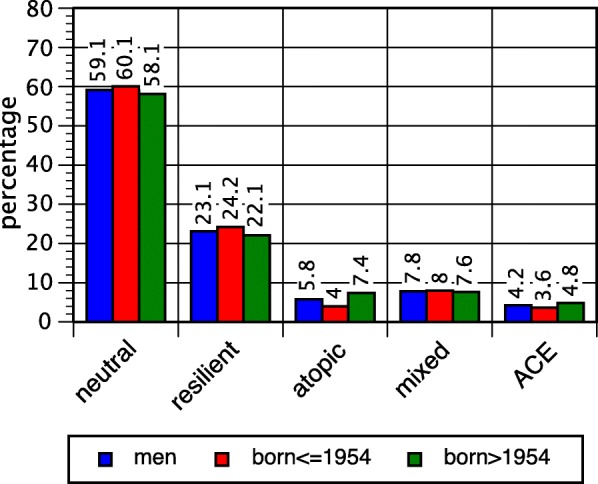
Proportions of latent classes, men; overall and after median split

**Fig. 5:**
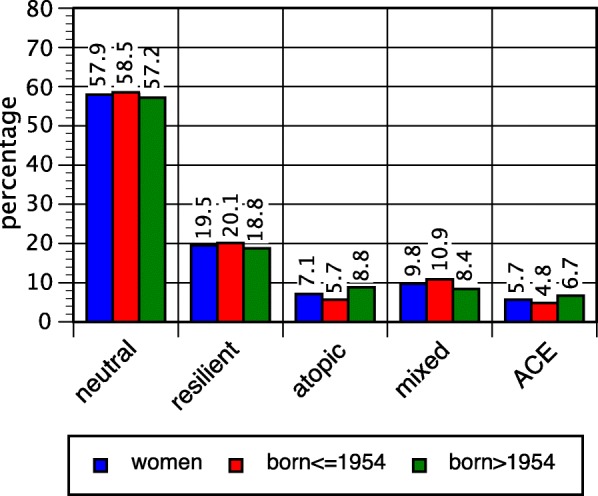
Proportions of latent classes, women; overall and after median split

The probabilities of each latent class are displayed in Fig. 2 (men) and Fig. 3 (women); the proportions of participants in the five classes are shown in Figs. 4 and 5. The results were largely congruent for men and women. The largest class comprised nearly 60% of participants. It was characterized by high probabilities regarding common childhood viral infections (chickenpox, measles, mumps), but low probabilities otherwise. The latter figures marked it as a baseline or neutral class. The second class comprised about 20% of participants for each sex. It stood out due to extraordinarily low probabilities in chickenpox/measles/mumps. Regarding other variables, this class displayed similar or even lower probabilities such as the neutral class. It was preliminarily labeled the resilient class. Classes 3–5 were smaller classes and included between 4 and 10% of subjects. Class 3 was characterized by atopic diseases (asthma, hay fever, eczema, urticaria, drug allergies). The probabilities in the atopic class as compared to the neutral class were lower regarding chickenpox/measles/mumps in women and mumps in men. Class 4 displayed increased probabilities of herpes labialis (fever blisters, i.e., herpes simplex) and childhood diseases other than chickenpox/measles/mumps (scarlet fever, pertussis, rubella). In addition, class 4 shared high probabilities in both eczema variables and in drug allergies. It was preliminarily labeled the mixed class. With respect to chickenpox/measles/mumps, the probabilities were lower in measles and mumps (men) as well as in mumps (women). Finally, class 5 was determined by ACE (men and women) and traumatic experiences in childhood (women). The further features of the ACE class entailed enhanced probabilities of herpes infections and hay fever (both sexes) and, in women, enhanced probabilities in other atopies. The ACE class displayed lower probabilities of measles and mumps (men) and of chickenpox/measles/mumps (women).

### Immune-mediated classes differ regarding socioeconomic and sociodemographic features

The size of each of the five classes was largely congruent in men and in women (Table 5, Figs. 4 and 5 (see overall values)). However, marked differences between the classes became apparent regarding socioeconomic status. The atopic and the mixed classes were consistently characterized by high average scores of the Hollingshead index in both sexes, and the ACE class (in men, also the resilient class) by low scores (see Table 1; in one-way ANOVAs *p* < 0.001).

**Table 5:** Sociodemographic and socioeconomic characteristics of immune-mediated classes, men and women

	Neutral class	Resilient class	Atopic class	Mixed class	ACE class	Overall
Men						
*N*	1337	523	131	177	96	2264
SES score	3.56 (3.50–3.63)	3.33 (3.23–3.44)	3.93 (3.73–4.13)	3.77 (3.60–3.94)	3.27 (3.00–3.54)	3.53 (3.48–3.59)
Mean age	53.6 (53.0–54.2)	54.4 (53.4–55.4)	50.1 (48.2–51.9)	53.3 (51.6–55.0)	51.5 (49.3–53.6)	53.5 (53.0–53.9)
Women						
*N*	1512	509	186	255	148	2610
SES score	3.19 (3.12–3.25)	3.20 (3.09–3.31)	3.39 (3.21–3.57)	3.44 (3.29–3.59)	2.95 (2.73–3.16)	3.21 (3.17–3.26)
Mean age	54.7 (54.2–55.3)	56.4 (55.4–57.5)	51.9 (50.2–53.5)	55.2 (54.0–56.5)	51.1 (49.7–52.6)	54.7 (54.3–55.1)

*ACE* adverse childhood experiences, *SES* socioeconomic status (Hollingshead index)

In both sexes, the mean age was distinctly lower in the atopic and the ACE classes than in the other classes. In order to gain insight into the historical change, a median split across the birth cohorts born in 1954 was applied (Figs. 4 and 5). The atopic and the ACE classes turned out to be more prominent in younger birth cohorts. In the atopic class, there was an increase from 4.0% in elder male birth cohorts to 7.4% in younger cohorts, and in women from 5.7 to 8.8%. The chi-square tests for the contingency tables (classes crossed with split birth cohorts) yielded 15.3 (d.f. 4, *p* = 0.004) in men and 18.4 (d.f. 4, *p* = 0.001) in women.

### Immune-mediated classes differ regarding WBC counts and inflammatory markers

**Fig. 6:**
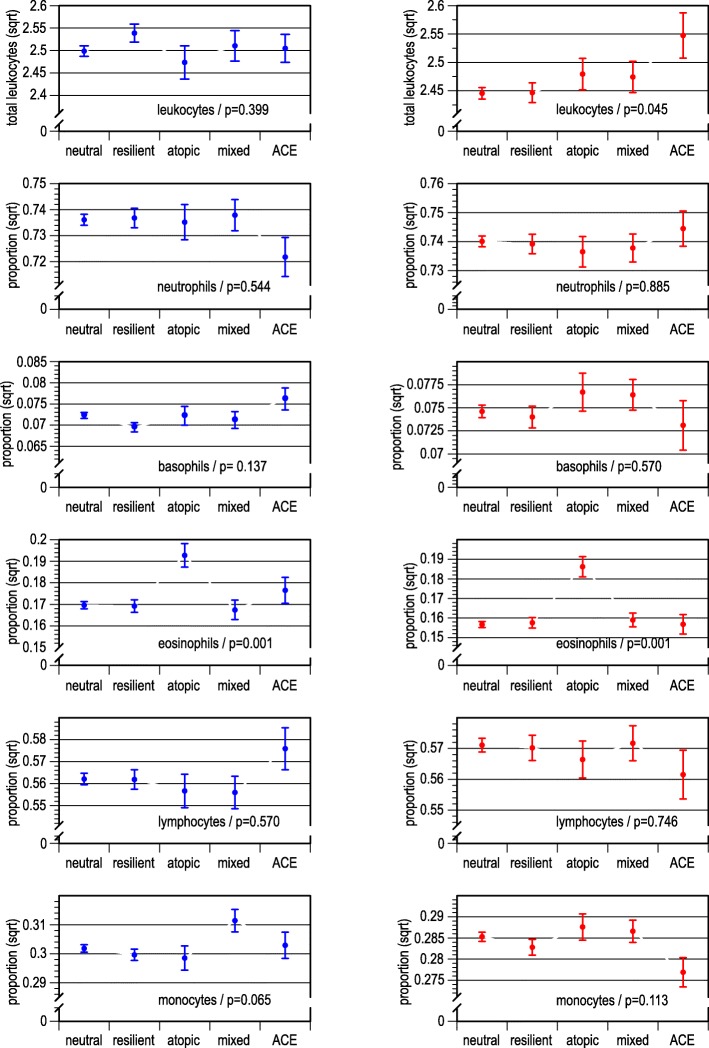
White blood cell counts across latent classes, by sex; means (leukocytes) and proportions (specific white blood cells); data smoothed by square root

Analyses of WBC counts (Fig. 6) with ANOVA returned as expected an increased proportion of eosinophils in the atopic class (class 3) both in men and women. In addition, in women, the overall leucocyte count was increased in the ACE class.

**Fig. 7:**
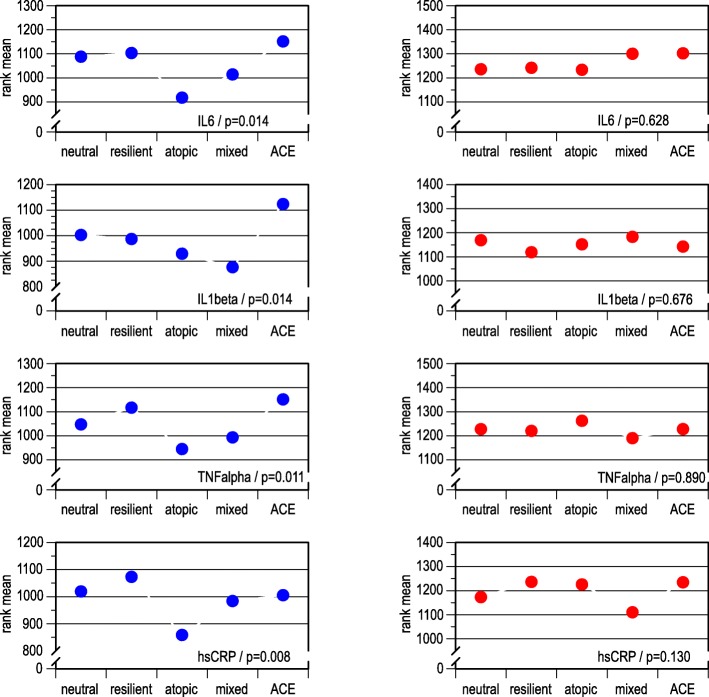
Mean ranks of inflammatory markers from two assessments, across latent classes, by sex

Kruskal-Wallis tests were applied to ranked data of inflammatory markers IL-6, IL-1β, TNF-α, and hsCRP (Fig. 7). In men, the atopic and the mixed classes consistently displayed lower rank means than the other classes. IL-1β was particularly high in the ACE class, but low in the mixed class—even lower than in the atopic class. In contrast to men, the results of the Kruskal-Wallis tests were not revealing in women.

### Immune-mediated classes’ associations with chronic inflammatory diseases and neurodevelopmental/mental disorders

Table 6 presents the odds ratios (ORs) of selected somatic conditions (lifetime prevalence) regressed on immune-mediated classes. Several patterns became apparent in the integrated perspective. The resilient class showed no (positive) associations with any of the conditions except with ulcer in men. This is a contrast to the atopic and the mixed classes, which had (positive) associations with several other conditions both in men and in women. The ACE class showed an additional pattern: associations with somatic conditions were prominent in women, whereas no associations were apparent in men. Thus, another aspect of sexual dimorphism becomes visible apart from inflammatory markers.

**Table 6:** Odds ratios (95% confidence intervals) of somatic diseases/disorders (lifetime prevalence) regressed on immune-mediated classes, men and women

	Neutral class	Resilient class	Atopic class	Mixed class	ACE class
Men					
Ulcer	1 (ref.)	1.6 (1.1–2.3)	1.0 (0.4–2.1)	0.7 (0.3–1.6)	0.7 (0.3–2.1)
Irritable bowel syndrome	1 (ref.)	1.0 (0.5–2.1)	3.0 (1.3–7.0)	2.5 (1.1–5.6)	1.1 (0.3–4.8)
Cystitis	1 (ref.)	0.7 (0.4–1.3)	3.2 (1.8–5.7)	3.3 (1.9–5.5)	1.5 (0.6–3.6)
Acne	1 (ref.)	0.7 (0.5–1.1)	1.4 (0.8–2.5)	1.8 (1.1–2.8)	0.7 (0.3–1.7)
Psoriasis	1 (ref.)	0.7 (0.4–1.1)	0.3 (0.1–1.2)	1.3 (0.7–2.5)	1.2 (0.5–2.9)
Migraine	1 (ref.)	1.1 (0.7–1.6)	1.8 (1.1–3.2)	2.3 (1.5–3.6)	1.3 (0.6–2.6)
Women					
Ulcer	1 (ref.)	1.3 (0.8–2.1)	1.5 (0.8–2.9)	1.4 (0.8–2.5)	3.1 (1.8–5.4)
Irritable bowel syndrome	1 (ref.)	1.5 (0.9–2.4)	2.0 (1.1–3.8)	2.9 (1.8–4.8)	3.7 (2.1–6.6)
Cystitis	1 (ref.)	1.0 (0.8–1.2)	2.1 (1.5–2.8)	1.9 (1.5–2.5)	2.2 (1.5–3.0)
Acne	1 (ref.)	0.9 (0.6–1.3)	1.1 (0.6–1.7)	1.5 (1.0–2.2)	1.8 (1.1–2.8)
Psoriasis	1 (ref.)	0.9 (0.5–1.5)	1.0 (0.5–2.1)	0.9 (0.5–1.8)	1.6 (0.8–3.2)
Migraine	1 (ref.)	0.9 (0.7–1.2)	1.7 (1.2–2.4)	1.2 (0.9–1.7)	1.2 (0.8–1.8)

*ACE* adverse childhood experiences

Additionally, sexual dimorphism is reflected on the level of specific disorders, i.e., migraine (in men associated with the mixed class in addition to the atopic class) apart from ulcer (see above). In contrast, irritable bowel syndrome and cystitis were similarly associated with the atopic and the mixed classes, and acne with the mixed class in men and in women. Psoriasis was the only disease without revealing associations with any of the classes.

The association patterns between the classes and major groups of neurodevelopmental/mental disorders (see Table 7) were basically similar to the somatic conditions. Sharing the resilient class yielded a consistent preventive effect against neurodevelopmental/mental disorders. In contrast, the ACE class was consistently positively associated with the latter—now, not only in women but also in men. The atopic and the mixed classes showed heterogeneous associations with neurodevelopmental/mental disorders which also varied by sex. The associations were strongest in the ACE class (ORs typically ranged 2–4), followed by the mixed class (1.5–2) and the atopic class (1–1.5).

**Table 7:** Odds ratios (95% confidence intervals) of groups of mental disorders (lifetime prevalence) regressed on immune-mediated classes, men and women

	Neutral class	Resilient class	Atopic class	Mixed class	ACE class
Men					
Neurodevelopmental disorders	1 (ref.)	0.8 (0.5–1.2)	1.7 (1.0–3.0)	2.0 (1.3–3.2)	4.0 (2.4–6.5)
Early anxiety disorders	1 (ref.)	0.8 (0.6–1.1)	1.1 (0.7–1.8)	2.0 (1.4–2.8)	2.7 (1.7–4.3)
Late anxiety disorders	1 (ref.)	0.7 (0.5–1.0)	1.2 (0.7–2.1)	1.5 (1.0–2.4)	1.6 (0.9–2.9)
Mood disorders	1 (ref.)	0.8 (0.7–1.0)	1.5 (1.0–2.2)	1.8 (1.3–2.5)	2.3 (1.5–3.4)
Substance disorders	1 (ref.)	0.9 (0.7–1.2)	1.1 (0.7–1.6)	1.1 (0.8–1.6)	1.8 (1.1–2.8)
Women					
Neurodevelopmental disorders	1 (ref.)	0.6 (0.3–1.0)	0.9 (0.4–1.8)	1.6 (0.9–2.6)	3.8 (2.3–6.2)
Early anxiety disorders	1 (ref.)	0.7 (0.5–0.9)	1.6 (1.2–2.2)	1.4 (1.1–1.9)	3.0 (2.1–4.2)
Late anxiety disorders	1 (ref.)	0.8 (0.6–1.1)	1.2 (0.8–1.8)	1.6 (1.1–2.2)	2.7 (1.9–4.0)
Mood disorders	1 (ref.)	0.9 (0.7–1.0)	1.1 (0.8–1.5)	1.4 (1.1–1.9)	2.2 (1.5–3.2)
Substance disorders	1 (ref.)	0.6 (0.4–1.0)	1.8 (1.0–3.0)	1.6 (1.0–2.6)	2.7 (1.6–4.6)

*ACE* adverse childhood experiences

Groups of mental disorders: neurodevelopmental disorders—tics, attention-deficit hyperactivity disorder, conduct disorder, oppositional defiant disorder; early anxiety disorders—separation anxiety disorder, overanxious disorder, specific phobias (animals), social phobia; late anxiety disorders—generalized anxiety disorder, panic, agoraphobia, specific phobias (excl. animals); mood disorders—major depression disorder, dysthymia, bipolar disorders; substance disorders—alcohol, cannabis, other illicit drug abuse/dependence

## Discussion

This is the first study to comprehensively determine immune-mediated classes that evolve early in life. LCA was applied on childhood infections, atopic diseases, and ACE which can be linked with immune system programming in childhood [2]. LCA determined five congruent classes in men and in women labeled according to the captured associations as neutral, resilient, atopic, mixed, and ACE classes. Further analyses aiming to describe these immune-mediated classes showed characteristic changes in WBC counts and inflammatory markers. In addition, the atopic, the mixed, and the ACE classes typically displayed positive associations with chronic inflammatory diseases and neurodevelopmental/mental disorders, whereas the resilient class emerged with mostly negative associations, i.e., suggesting a protective relation. The classes differed with respect to socioeconomic status and birth cohort. The following paragraphs will discuss the most important patterns and findings.

### Characterizing the immune-mediated classes

The five classes derived from PsyCoLaus data represent the maximum solution which is appropriate with this data set. For theoretical reasons (e.g., heterogeneity of asthma and other atopic diseases) and for empirical reasons (e.g., no associations involving psoriasis in this study), a higher number of classes would be expected, but could not be achieved due to the limitations of the sample size, the variable configuration, and the statistical model.

The neutral class with more than a half of all subjects was considered as the baseline class. This implied that all other classes separate from the neutral class during development. The latter provided the baseline prevalence rates of childhood infections and atopies and the reference class for further analyses. While most prevalence rates of childhood infections and atopic diseases were low in this class, the rates of chickenpox, measles, and mumps were particularly high, thus signaling the absence of any extraordinary immune system activity. Subjects pertaining to the neutral class displayed low prevalence rates in chronic inflammatory diseases and neurodevelopmental/mental disorders.

The resilient class shared core features with the neutral class such as the low prevalence rates of childhood infections and atopies. However, it displayed outstandingly low rates with respect to chickenpox, measles, and mumps. This was particularly noteworthy, given the high infectiousness of chickenpox and measles, as well as their seroprevalence of antibodies and reporting rates of above 90% before the start of vaccination campaigns in the 1960s and 1970s. Another outstanding feature of this class and a potential clue to its interpretation was the positive association with ulcer (men). *Helicobacter pylori*, the most important risk factor in ulcer [46, 47], is one of the “Old Friends” within the framework of the hygiene hypothesis [4], thus indicating that the resilient class in fact represents the “Old Friends” class. On the outcome side, this class was related to consistently lower risk regarding all groups of mental disorders, both in men and in women. This is in agreement with the hygiene hypothesis which postulated that microbial and helminthic pathogens (the “Old Friends”) encountered in infancy and early childhood can train the immune system and improve its capacity for inflammation control and thus decrease the susceptibility for depression and other mental disorders [9, 48].

The atopic, the mixed, and the ACE classes were marked by increased risk for chronic inflammatory diseases and neurodevelopmental/mental disorders. Obviously, the atopic class was characterized by high probabilities for all atopies. The proportion of eosinophils in WBC counts was increased, whereas, in men, the levels of inflammatory markers were consistently lower than those in other classes. The atopic class included enhanced risk for irritable bowel syndrome, cystitis, and migraine [49]. It was associated with mental disorders both in boys/men and in girls/women. This is in line with a large amount of comorbidity studies reporting associations between atopic diseases and neurodevelopmental/mental disorders [50–61].

The mixed class revealed an additional and new perspective, while all other classes could have been hypothesized in advance based on available research results. It relied on a mix of specific atopies (eczema, drug allergies), childhood infections other than chickenpox/measles/mumps, and herpes simplex (herpes labialis), which is also typically a childhood infection. Similar to the atopic class, the mixed class showed lower levels of inflammatory markers in men, however, intriguingly no increase of eosinophil counts. Another parallel with the atopic class derived from the associations with socioeconomic status: both classes were more prevalent in the middle and upper classes than in the lower classes (for the atopic class, see [62]). Members of this class had increased risk for irritable bowel syndrome, cystitis, acne, and migraine (men). Compared to the atopic class, the mixed class displayed overall higher ORs for neurodevelopmental/mental disorders.

Among all classes, as an expected finding, the ACE class showed the highest associations with neurodevelopmental/mental disorders. Apart from that, there was a multitude of interferences with somatic conditions: childhood infections (lower reporting of chickenpox/measles/mumps), atopies (higher frequencies of hay fever, and, in women, other atopic diseases), chronic inflammatory diseases (only women). The latter finding suggests that ACE trigger stronger imbalances of the immune system in girls/women than in boys/men.

### Implications for changing incidence/prevalence rates of atopic diseases and mental disorders

The increase in prevalence rates of asthma and other atopic diseases has been a major issue in public health in recent years. This increase is assumed to have been most accelerated between the 1960s and 1990s [63], and there is evidence that it was stronger in girls/women than in boys/men [64]. A similar discussion has taken place in public mental health regarding increasing rates of depression [9, 65]. The present analysis provides a new perspective for these discussions by comparing the changes within the classes across birth cohorts. As expected, we found a higher proportion of the atopic class in younger birth cohorts, which occurred at the expense of the resilient class and the mixed class. The consequences must be more complex than outlined by the “Old Friends” hypothesis. While the changes within the atopic and the resilient classes together contribute to an increasing risk for chronic inflammatory diseases and neurodevelopmental/mental disorders, this shift is counterbalanced by a decrease in risk for diseases associated with the mixed class.

### Time of onset of childhood infections and timing issues

The interplay between the neutral class and chickenpox/measles/mumps provides a suggestion to understand the timing, when immune-mediated classes separate from the neutral class. Chickenpox/measles/mumps are expected to yield seroprevalence rates of antibodies and reporting rates of about 90% under unambiguous conditions before the start of vaccination campaigns in the 1960s and 1970s. Such reporting rates were consistently present in the neutral class but not in the other classes. Therefore, lower chickenpox/measles/mumps probabilities are presumed to signal some specific immune system activity limiting or influencing the duration and manifestation of chickenpox/measles/mumps infections. Furthermore, chickenpox/measles/mumps and other childhood infections with self-reported age of onset between 6 and 8 years [66] provide preliminary reference time points (in comparison with that: pertussis 6.5 years, rubella and scarlet fever 7.5 years; these data represent the upper age limits due to telescoping effects in reporting remote events (see also reference [67])).

By far, the lowest probabilities in chickenpox/measles/mumps compared to the neutral class emerged in the resilient class. Research on the “Old Friends” and the microbiota hypothesis has suggested that the resilient class might emerge as early as during the neonatal period [3, 68]. Marked deviations from the neutral class pattern also occurred in the ACE and the atopic classes in women, therefore raising the question whether these classes evolve at an earlier age in girls than in boys. Still, the mixed class (both sexes) and the ACE and atopic classes in men displayed lowered probability of mumps which means that they at least started to evolve at the same age as the age of mumps onset.

The differentiation of immune-mediated classes might end by adolescence, as indicated by migration studies in multiple sclerosis [69] and in depression [9]. Many further questions might be open to debate, e.g., are class changes and different sequences of class membership possible? If yes, how do they take place and what conversion probabilities do they comprise? Findings suggesting that low-level microbial exposure in infancy is protective against the negative outcomes of psychosocial stress such as increased CRP levels [65, 70] at least shed some light on the resilient class.

### Strengths and weaknesses

The immune-mediated classes yield many new perspectives that could not be discussed in more detail due to space constraints. Research on complex and heterogeneous issues such as the interrelations between the immune system, chronic inflammatory diseases, and mental disorders requires a comprehensive data background and appropriate analytical approaches. The LCA served pattern recognition and provided us with more details than variable-oriented models would have done.

However, LCA delivers exclusive classes, which is a simplification of the reality in most instances. LCA strongly depends on data input, selection of variables, sample sizes, and other limitations of the study. The analysis included only infectious and atopic diseases, chronic inflammatory diseases, and neurodevelopmental/mental disorders with sufficient frequencies (*n* > 50 in each sex) in the CoLaus|PsyCoLaus study. The CoLaus|PsyCoLaus study comprises only a limited number of variables covering psychosocial adversities. We cannot exclude the possibility that additional immune-mediated classes exist. Many subtypes of important chronic diseases and neurodevelopmental/mental disorders probably could not be covered by the classes presented here. For example, several subtypes of asthma are known [71], but they were not mirrored in the current analysis.

In addition, the range of inflammatory markers that served to characterize the classes was limited. The selection focused on markers that were intensively discussed at the beginning of the study in the early 2000 years. In the meantime, the range of inflammatory markers of interest has grown and includes both anti-inflammatory markers (e.g., IL-10) and pro-inflammatory markers (e.g., IL-8, IL-23). It is left to subsequent studies to examine their interplay in the different classes.

This study shares further common limitations of studies based on self-reporting data, including recall bias and telescoping effects [67]. In PsyCoLaus, many infections and pathogens of interest were not comprised in data due to limitations of self-reporting. Regarding adverse experiences and stigmatized issues, underreporting is probable and results in underestimation of statistical effects. Using groupings of neurodevelopmental/mental disorders, as well as chronic inflammatory diseases, which typically subsume different subtypes, has a further smoothing influence on statistical effects.

## Conclusions

Associations between childhood infections, atopic diseases, and adverse childhood experiences reveal a system of immune-mediated classes that incorporates and goes beyond the resilient (or “Old Friends”) class related to the hygiene hypothesis. The immune-mediated classes consolidate during the first years of life. Sharing a specific immune-mediated class different from the neutral class (resilient, atopic, mixed, or ACE class) modifies the risk for most chronic inflammatory diseases and neurodevelopmental/mental disorders. In particular, the mixed class emerged as a new and unanticipated issue. A better understanding of the immune-mediated classes provides a preliminary basis from which to start assessing more specific immune mechanisms relevant to particular chronic inflammatory diseases, neurodevelopmental/mental disorders, and their subtypes.

## Abbreviations

ABIC: Sample size-adjusted BIC
ACE: Adverse childhood experiences
AIC: Akaike information criterion
BIC: Bayesian information criterion
DIGS: Diagnostic Interview for Genetic Studies
hsCRP: High-sensitivity C-reactive protein
IL-1β: Interleukin-1β
IL-6: Interleukin-6
LCA: Latent class analysis
LMR-LRT: Lo-Mendell-Rubin likelihood ratio test
OR: Odds ratio
TNF-α: Tumor necrosis factor-α
WBC: White blood cell
